# Digital mental health interventions for chronic serious mental illness: Findings from a qualitative study on usability and scale-up of the Life Goals app for bipolar disorder

**DOI:** 10.3389/fdgth.2022.1033618

**Published:** 2022-11-21

**Authors:** Amy Rusch, Isabel Carley, Pratiksha Badola, Celeste Liebrecht, Melvin McInnis, Kelly A. Ryan, Shawna N. Smith

**Affiliations:** ^1^ Department of Health Management and Policy, University of Michigan School of Public Health, Ann Arbor, MI, United States; ^2^ Department of Psychiatry, University of Michigan Medicine, Ann Arbor, MI, United States; ^3^ Department of Learning Health Sciences, University of Michigan Medicine, Ann Arbor, MI, United States

**Keywords:** mental health, DMHI, bipolar disorder, qualitative study, affinity mapping, self-managament

## Abstract

The Life Goals (LG) application is an evidence-based self-management tool intended to help individuals with bipolar disorder (BD) by aligning symptom coping strategies with personal goals. The program has traditionally been offered in-person or *via* the web, but has recently been translated into an individualized, customizable mobile intervention to improve access to care and reduce provider burden. The LG app previously showed acceptability with ease of use and satisfaction with user interface, but less success in encouraging self-management. To better understand patient needs, our team conducted semi-structured interviews with 18 individuals with BD who used the LG app for 6 months. These interviews also investigated participant interest in sharing LG app data with their provider through an online dashboard. Using affinity mapping, a collaborative, qualitative data analysis technique, our team identified emerging common themes in the interviews. Through this process, team members identified 494 pieces of salient information from interviews that were mapped and translated into three main findings: (1) many participants found Mood Monitoring and LG modules helpful/interesting and stated the app overall had positive impacts on their mental health, (2) some components of the app were too rudimentary or impersonal to be beneficial, and (3) feedback was mixed regarding future implementation of an LG provider dashboard, with some participants seeing potential positive impacts and others hesitating due to perceived efficacy and privacy concerns. These findings can help researchers improve app-based interventions for individuals with BD by increasing app usage and improving care overall.

## 1. Introduction

Digital mental health interventions (DMHIs) are promising avenues for improving equitable access to mental health support while providing increased flexibility and opportunities to engage in healthcare management for individuals with mental illnesses. App-based mental health support can address barriers that prevent individuals from seeking care, such as access, cost, and stigma, and provide greater flexibility for long-term care (1–3). The use of DMHIs for mental health also has potential to alleviate pressures facing providers, including mental health provider shortages and a reliance on examination and testing, further increasing availability of care and bridging between provider appointments for patients with chronic needs (4, 5). The increased attention devoted to this topic in recent years, including DMHI implementation and scaling, highlights the potential that DHMIs have for meeting an increasing demand for mental health care (4, 6–8).

While DMHIs hold promise as mechanisms for treating mental illness and promoting mental well-being, multiple barriers exist to the regular and/or standardized use of DMHIs as a treatment modality (9). These barriers include technology-related difficulties (e.g., apps crashing), costs associated with app usage (e.g., need for smartphone, internet costs), privacy and confidentiality concerns, user engagement, and others (8, 10–12). Concerns around the ability (or lack thereof) of DMHIs to provide the same support as face-to-face treatment, tailor content to users unique needs, and respond to mental health crises also persist (6, 13). While DMHIs may provide more equitable access to care, paradoxically, some worry that reliance on DMHIs could lead to a fortification of mental health inequity as opposed to reducing it for those unable to reliably utilize DMHI technology or potentially affected by changes to the behavioral health landscape in response to growing DMHI use (3, 4).

To better design DMHIs for individuals with mental health illnesses, it is important to understand which features resonate with users and which additional supports they desire. DMHIs that exist for individuals with Bipolar Disorder (BD) include internet-based and smartphone interventions that provide information and/or management tools for screening and assessment, symptom monitoring, and community support. However, many of these are not grounded in research, lack validated assessment/screening measures, and are not in line with practice guidelines or established self-management principles (14). Further, especially for conditions like BD that are chronic and lifelong, it is important to understand how to adapt provision of DMHIs, both within users, as their need for support changes over time, as well as across users, recognizing that the needs of newly-diagnosed BD patients are likely different than those with experience managing their diagnosis and symptoms (15).

### 1.1. Life Goals

The app used in this study, Life Goals (LG), is based on the Life Goals Collaborative Care (LGCC) program, an evidence-based self-management intervention traditionally delivered in person or *via* the web, designed to assist individuals with BD by aligning symptom coping strategies with their personal goals (16, 17). In the LG app, described below, users are presented with various self-paced modules on mental health and wellness, feedback that is customizable to the user, and components to monitor their mood. Initial translation of the LG program into an app was previously rated feasible and acceptable by users with BD (25/28; 89%), but fewer participants found the content helpful for managing their health or in making progress towards their wellness goals (18).

This report discusses the findings from a qualitative evaluation focused on understanding the user experience from a group of pilot LG users. Specifically, our evaluation focused on strengths, barriers, and areas for improvement. Additionally, we solicited user opinions on the perceived risks and benefits of a technology-mediated method of sharing app data with providers *via* a dashboard.

### 1.2. Life Goals: Smartphone application

**Figure 1:**
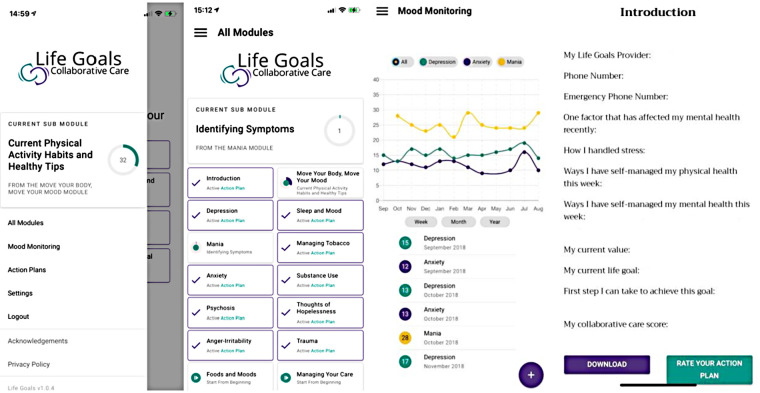
Life Goals app interface.

The LG app (Figure 1) includes succinct, 5–10-minute-long modules that provide activities for self-management of BD. The content and wording of the modules were adapted from the original LGCC program (16, 17). Modules (Figure 1, Panel 2) provide participants with instructions on identifying symptoms and triggers for different moods and behaviors, tips for goal setting and encountering roadblocks, and information on how different reactions may impact participants' overall values and life goals. For each module, participants can select which symptoms and triggers they have struggled with prior. Upon completion, their answers are added to their personalized “Action Plan,” which combines participant answers entered throughout the module into a chart that they can refer to for help effectively manage their symptoms. The Action Plans (Figure 1, Panel 4) outline the participant's personal experience and how they can use their own healthy responses to prevent or limit negative moods and/or behaviors, specific to each module (i.e., Mania, Depression, etc.).

The Mood Monitoring component (Figure 1, Panel 3) enables participants to monitor their symptoms of anxiety, depression, and mania using standardized measures (19–21). Participants receive an automatically calculated mood rating displayed in a graph, and are able to view trends in their moods over the course of a week, month, or year.

### 1.3. Life Goals: Provider dashboard

**Figure 2:**
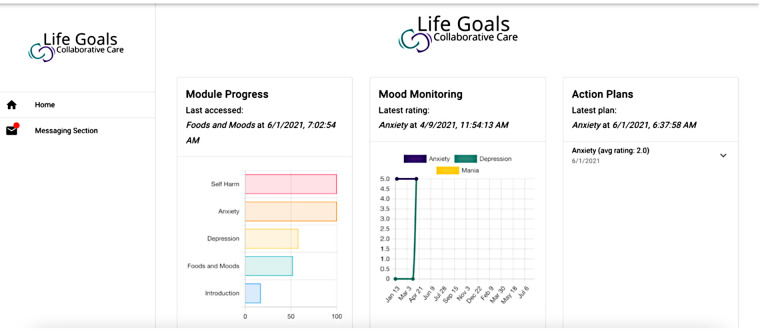
Life Goals provider dashboard home screen.

The provider dashboard (Figure 2) was developed as an augmentation to the patient-facing LG app for use by providers of patient-participants using the LG app. The dashboard was conceptualized to relay important metrics to providers, including their patients' module progress, Mood Monitoring scores, and Action Plans to keep providers informed, potentially convey warning signs (of mania, depression, medication non-adherence), and help better adapt patient treatment.

## 2. Methods

Individuals were recruited to use the LG app from the Heinz C. Prechter Longitudinal Study of Bipolar Disorder cohort at the University of Michigan (22). Potential participants were invited to participate *via* email sent by study staff and enrollment into the LG study began in May 2018 and concluded in May 2021. Participants were included if they were 18 years or older, had any BD diagnosis [i.e., Bipolar I, II, or not otherwise specified (NOS)], were community dwelling (i.e., not living in a nursing home or other institution), and owned a smartphone. Exclusion criteria were any serious illness precluding participation in LG components or inability to provide informed consent.

Participants were provided with instructions on downloading and using the LG app on their own smartphones, then instructed to use it as they felt beneficial (i.e., without any stated dosage or time requirement) for the 6-month duration of the study. Participants were required to complete the Introduction module, then to utilize the modules they found most applicable to their personal experience with BD; they could access as few or as many modules as desired. A majority of our sample did not have prior experience with DMHIs. Further information about the original LGCC program and initial development/usability of the LG app can be found elsewhere (16–18).

For this qualitative follow-up study, all participants who enrolled in and completed the larger LG study were invited to complete a one-hour interview with study staff over Zoom to provide feedback about their experience with the app. Interviews were conducted August-October 2021. Interviewers asked participants to reflect upon their experiences using the LG app and share the components that were most helpful to their care using a semi-structured interview guide created by senior study team members. The interview guide probed on participant engagement (frequency, duration), feature usage (Mood Monitoring, modules), and overall satisfaction with the app. Participants also provided suggestions for refinements and additions to the app, as well as thoughts on the provider dashboard. Participants were compensated $20 for participating in the interview. This study was approved by the University of Michigan Institutional Review Board, and all participants provided signed informed consent.

### 2.1. Affinity mapping

**Figure 3:**
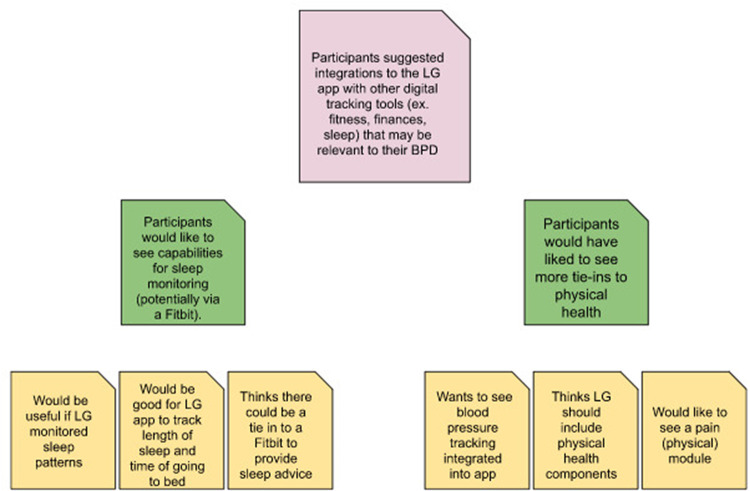
Example of the affinity mapping process.

Affinity mapping was used to conduct our analysis. Also known as affinity diagramming or the Kawakita-Jiro (K-J) Method, affinity mapping is an inductive, bottom-up process performed collaboratively by a team to synthesize qualitative data into thematically related groups without using predetermined categories (23, 24). Affinity mapping has been used successfully as a tool for thematic analysis of qualitative data in previous research as an agile, cooperative technique based on inductive reasoning to efficiently group data without preexisting parameters (25–27). Upon completion, interviews were transcribed verbatim by study team members. Each team member independently reviewed the transcripts to obtain excerpts of salient information that encapsulated one unique point shared by those being interviewed. These excerpts were compiled in an Excel file, then exported to an online collaboration platform (Miro.com) to create virtual sticky notes. Over multiple affinity mapping sessions, team members collaboratively reorganized the sticky notes based on content, grouping together similar excerpts to create thematic categories (Figure 3).

## 3. Results

*N* = 91 individuals with BD participated in the larger study piloting the LG app for 6 months. Although results from this full set of users are still pending, results from an early subset of users have been previously published and found higher user satisfaction, but heterogeneous engagement that declined over time (18). All *N* = 91 users were invited to participate in this follow-up qualitative study; *N* = 79 were contacted in July 2021; another 12 participants were contacted in September 2021, after they completed their six months of LG app usage. Overall, *N* = 18 (20%) agreed to an interview. The majority had a diagnosis of Bipolar 1 and identified as female; mean age was 49.2 and mean time since diagnosis was 33.4 years (Table 1).

**Table 1:** Participant demographics from Life Goals interviews.

Variable	*N* = 18 (%) / M (SD)
Age	49.2 (13.7)
Length of diagnosis in years	33.4 (14.0)
Minimum	11
Maximum	52
Gender	
Female	12 (66.7%)
Male	6 (33.3%)
BP Diagnosis	
BP I	15 (83.3%)
BP II	2 (11.1%)
BP-NOS	1 (5.6%)

### 3.1. Affinity mapping findings

Upon initial review of the interview transcripts, 494 excerpts of salient information were identified, ranging from short phrases summarized from the interviews to full quotes from participants. Quotes were used when multiple interpretations could be gleaned from excerpts and were discussed by team members throughout the affinity mapping process to ensure meanings were categorized correctly. The team first grouped the excerpts into base categories, then identified clusters of those categories that were thematically similar to identify main ideas seen across interviews. Main ideas were then thematically combined to yield three major findings relating to the user experience of the LG app.

#### 3.1.1. Finding 1: Participants had positive experiences with Mood Monitoring and modules, finding them helpful and/or interesting, and believed the app had positive impacts on their mental health

##### 3.1.1.1. Mood Monitoring

Participants felt that an awareness of their mood over time was important to their self-management and that this process was enhanced through the Mood Monitoring feature. One participant shared that the LG app was helpful in that it made them more “conscious and aware” of what was going on with their mood. Other participants noted the monitoring component was especially helpful when experiencing manic or hypomanic episodes (i.e., racing thoughts, distractibility) as those are times when they are less likely to be aware of how they are feeling.

Participants also liked that Mood Monitoring helped them identify patterns and issues relating to their mental health. For example, some participants noted a relationship between sleep and mood that had been harder for them to identify before using the feature. Some used Mood Monitoring as a “warning sign” to prepare for mood and behavioral changes stemming from their BD. One participant stated: “Warning signs may not stick out day-to-day, so having an overall view of [my] mood could be helpful to track if [I’m] getting more depressed or elevated.” Another participant noted that they appreciated the Mood Monitoring graph as it was helpful to “visualize mood trending upwards or downwards over time”. Participants liked that Mood Monitoring was quick and easy to use. When asked about their favorite app feature, one participant stated, “The Mood Monitoring. It was simple, it didn’t take a lot of time, and I could watch my mood over a period of time.”

##### 3.1.1.2. Modules

Overall, participants found the module content interesting and relevant to their mental health needs. Participants generally relayed positive experiences completing the LG modules and appreciated that they could revisit specific modules if there was a certain topic that was particularly salient for them at any point, and as often as they liked, over the six months of the study. Preferences towards specific modules were driven by personal experiences or needs. For example, one participant looked to the trauma module to learn more about how a past traumatic event may have affected him and his mental health. Preferences also varied by module use, which interviews revealed to be heterogeneous among participants, with some reporting frequent and consistent use while others disengaging quickly.

##### 3.1.1.3. Action Plans

Participants did not provide much feedback regarding the Action Plans, although one participant noted that they really liked the goal-setting aspect of the app and how the Action Plans were broken down into the categories of the corresponding modules (e.g., Substance Use, Depression). Another participant noted they did revisit their Action Plans, but their use of the plans tapered off throughout the study. A third participant noted they utilized the emergency contact feature of the Introduction Action Plan. The Action Plan feature was developed in spring 2021, but pushed into production as an update to the app in fall 2021, after many participants had completed the study; this may be why there is generally less feedback on this feature.

#### 3.1.2. Finding 2: Participants felt components of the app were too rudimentary or generic to be beneficial or applicable to them and wanted to enhance customizability and interactivity of the app

##### 3.1.2.1. Mood Monitoring

A major criticism of the Mood Monitoring component was that it was too generic and did not address certain behaviors uniquely relevant to participants when monitoring their mood. One participant remarked, “I wish that it would have been better at tracking my mood. It seemed like there were a few general topics, but it didn’t get into the specifics of, ‘Have I been spending too much money?’, ‘Am I staying home too much?’, Specific things that would let me know that there's something going on that I need to pay attention to.” This speaks to a desire to monitor behaviors that are more personally relevant to how BD manifests itself, which would thereby provide more accurate insights. For example, participants felt medication tracking could be a beneficial tie-in to Mood Monitoring. Financial tracking was another common suggestion, as finances are, as one participant put it, an area where they were more likely to “go off the radar.” Further, participants would like the LG app to incorporate aspects of physical health such as physical pain, hormone-tracking, and diet. One participant suggested creating “a more tailored user experience” by integrating Fitbit data to the LG to provide physiological data on sleep quality and activity levels alongside Mood Monitoring.

A few participants felt the Mood Monitoring component of the app was repetitive. One participant said, “it came to the point where the Mood Monitoring was my least favorite [feature] because it just was so redundant with the same questions.” This participant went on to suggest that changing the order of the questions daily could help to minimize the repetitive nature of this feature. As the questions are based on clinical diagnostic questionnaires (e.g., PHQ-9, GAD-7), changing the content or ordering of the questions may affect the previously determined reliability and validity of those questionnaires (28); regardless, it is worthwhile to note that the reaction to the repetitiveness is strong enough that it may discourage participant use of the feature entirely. To note, these validated scales are not typically designed for frequent use, so understanding how these measures could be adapted or shortened over frequent use is an important question for DMHI researchers to consider to ensure accurate and consistent engagement over time.

##### 3.1.2.2. Modules

Although participants liked that module topic selection could be tailored to their needs, they also noted that module content was often generic and not tailored to unique user needs. Some participants felt many modules were not applicable, or that the modules were rudimentary in terms of the content that was covered. These comments likely reflected that many of our participants had lived with BD for some time (decades, on average), and thus had established expertise on their condition. Commonly, participants noted the existing app modules may have been more beneficial to them earlier in their course of diagnosis. A participant stated, “I didn't really use much of the modules …I’ve been diagnosed for a long time, and so I feel like I have a good sense of what my diagnosis entails. So, it could be useful for somebody who's just starting on their journey.” Another participant said, “[Modules] didn’t tell me anything that I didn’t already know. The recommendations were so basic and the way they were described was simplistic … For me who's lived with [BD] for 10 years or longer, it just didn’t seem helpful.”

To remedy this and allow for greater tailoring to content level, this same participant suggested, “[The LG app] could have an introductory questionnaire, so you can see how long you've been struggling with [BD] and how much your need is for this kind of support, how much you know about coping skills, and how much you know about your moods.” Similarly, another participant suggested having different “tracks” (i.e., beginner, intermediate) on the app, based on how experienced users are with their BD diagnosis.

Another suggested developing an algorithm that recognizes what components the LG app user is interacting with and feeds them personalized modules based on their activity. This participant also suggested adding modules to the app over time, so users pace themselves better and continually have novel content to engage with. Regarding the pacing of the modules, someone suggested “If you're going to make an app that helps, it's got to work in the same way a counselor would work. They don’t slam you with everything in those first six months. They’ve got to get to know you to tailor the help they’re trying to give.”

##### 3.1.2.3. App suggestions

Participants made other suggestions to increase the interactivity of the app, for purposes of more engaged and consistent usage. Some participants suggested they would like the LG app to allow them to interact and share their experiences with others using the app because that would increase their engagement. One participant suggested that the addition of a chat forum to the app where “people could go in privately, anonymously, and talk to each other about what they're struggling with and share coping skills” would be welcome, as this would allow participants to share their experiences and feelings, reduce stigma, and normalize living with BD.

While some participants asked for more opportunity to interact with other users, others wanted the app itself to be more engaging or novel across time. Many participants suggested that they would like to see regular and/or varied prompts from the app to stimulate their use and engage them to promote more consistent usage. Similarly, one participant suggested the app could provide mental and physical health “tips-of-the-day” to increase daily engagement with module use and mood tracking. Others suggested tailored opportunities to promote more regular engagement with the LG app. To demonstrate this, one participant suggested programming the app to allow users to program a quote or saying that they found helpful or uplifting to encourage usage over time.

Multiple participants suggested personalized feedback provided by the app based on their responses. For example, one participant suggested adding a journal feature, in which the participant could receive thoughtful feedback from a provider or trained professional based on their entries. This participant hoped that feedback would “not just be like ‘good job,’ but something where someone actually reads it, understands it, and gives insightful information about it.”

#### 3.1.3. Finding 3: Some participants felt a provider dashboard could have positive impacts and were comfortable with providers viewing their data continuously, while others had hesitations with the provider dashboard due to perceived efficacy and/or privacy

Participants were mixed in reaction to development of a LG provider dashboard. This tool is currently being developed as a component of the LG app with the intent to inform a learning mental health care system (29–31) by allowing providers access (with patient permission) to patient data between appointments, for example on Mood Monitoring or module engagement, to better understand their patients' symptom trajectories and goals and subsequently adapt treatment and improve outcomes.

Participants had mixed feelings in their hypothetical comfort level with using a digital dashboard to share mood-related data with their mental health providers. Some felt the use of a provider dashboard would be helpful and have positive impacts. One participant, for example, saw the dashboard as beneficial as it would improve the interactivity of the LG app and feed into tailored feedback: “[T]hen at least I don’t feel like my information is going to empty air, that there's some type of receiving end to it.”

Multiple participants also shared that they would be comfortable with their providers viewing their data *via* a provider dashboard between visits. One participant wanted her providers to have an unrestricted view of her LG data on their dashboard. Another participant postulated that it would be helpful for his psychiatrist to routinely check his dashboard (e.g., once a month) to see what, if anything, had been changing with his mental health since he only sees his psychiatrist once a year.

However, some participants had hesitations with the provider dashboard due to concerns about privacy or provider interest/use. One participant felt the dashboard was too invasive: “This [feature] would need to be optional; I'd just feel like Big Brother was watching me.” Other participants didn't believe their providers would look at the data provided, negating the perceived effectiveness of this tool.

A subset of participants were generally open to sharing LG data *via* the provider dashboard but felt there should be limitations as to what providers could access. Participants indicated that they would want to opt in or out of sharing data with providers *via* the dashboard, and/or for provider dashboard access to be “renewed” or revocable regularly (i.e., *via* electronic consent form).

## 4. Discussion

Identifying salient findings from interviews using affinity mapping methodology has the potential to improve the usability of the LG app and others like it for those with BD and other mental health concerns. Understanding which features most resonate with participants allows DMHI creators to better engage patients *via* technology-mediated interventions and sustain their use long-term. Understanding factors participants didn't like or which precluded sustained engagement with the LG app also highlights promising areas for improvement, especially when coupled with participant's expressed interest in using LG long-term. These findings, and especially suggestions for enhanced customizability and interactivity, illuminate opportunities to improve the LG app and may also be generalized for improving other DMHIs targeting BD or other similar mental health concerns.

Participant requests for improving the LG app often involved more personalized feedback and/or integration with other aspects of their health. These comments reflected a desire for greater awareness and monitoring of other behaviors that are less explicitly tied to their mental health but are nonetheless relevant given their personal manifestation of BD, and thus can provide more accurate insights into how they are *personally* faring. These requests reflect downstream needs for individuals with BD, including a need for DMHI researchers and developers to understand how to creatively personalize and engage users with digital mental health support. Given the personal nature of this tailoring, however, designing systems that ensure consumers have control of how much or what is personalized or customized (e.g., through options to opt-in) should be concurrently prioritized.

These considerations are also crucial for developing just-in-time, adaptive interventions (JITAIs) in the BD space (32). JITAIs are a type of intervention that provides tailored support at the exact time of need and JITAIs' utilization of mobile technologies to capture the changing states of individuals aids in the development of personalized and real-time intervention strategies (32–34). Understanding users receptivity to potential customization in DMHIs *via* JITAIs holds promise for advancing engagement and satisfaction in this space. This work also adds to research showing that personalized feedback and support often leads to greater engagement with DHMIs in the mental health space and furthers researcher and developer understandings of user motivations for wanting personalization and/or tailoring and why they may matter for supporting longer-term DMHI adherence or engagement. Our findings revealed an array of preferences toward personalization, interaction, and feedback provided by the LG app and future work understanding these nuances is vital for DMHI success. For example, such insights can help scaffold understanding of personalization “do's” and “don’ts” in the DMHI space, which is critical for prioritizing high-quality data capture and integration for tailoring while also protecting user privacy and minimizing user burden.

### 4.1. Limitations

The study included a small sample size of participants who were both part of the Prechter Longitudinal Study and had previously participated in a study using the LG app. As such, these participants are likely more aware of their current needs and more interested in pursuing treatment through DMHIs than other individuals with BD. Further, participants had only engaged with the LG app for up to six months, limiting their perspectives on long-term LG app use. Additionally, interviews were retrospective. As enrollment into the original study and subsequent use of the LG app occurred over a two-year period, participants no longer had access to the app at the time of the interview, thus participant recollection (and subsequent specificity) varied. The sample was predominantly white, non-Hispanic females in middle age (M = 49.2 years old), which limits the generalizability of our findings. Further, the average age of our sample was about 19 years above the usual age of onset for BD, indicating that our participants may have been living with BD for a longer period and therefore, these results may not be applicable to individuals early in their course of illness.

### 4.2. Future directions

These findings will be used to inform the development of future DMHIs and app-based mental health support. Specifically, this work will guide next-round developments for the LG app and determine where and how best to use the LG dashboard to bridge the gap between BD patients and providers in monitoring BD symptoms, progression, and any relationships with LG app usage. Other work has highlighted the benefits of implementing DMHIs in health care settings to improve system integrations and communication and further reduce the research-to-practice gap (7, 35). The feedback provided by individuals with BD on the LG app and dashboard will aid in the effective scale-up of both this intervention and others to provide more accessible and interactive mental health support through digital mental health interventions.

## 5. Conclusions

DMHIs have the potential to meet the increasing demand for mental health care by providing flexibility for engaging in healthcare management, thus improving equitable access to mental health support for those with BD and other mental illnesses. Understanding the features of DMHIs that most resonate with users while acknowledging the current factors that are not as favorable will allow DMHI researchers and developers to improve the state of the technology, thus increasing usage.

## Data Availability

The raw data supporting the conclusions of this article will be made available by the authors, without undue reservation.

## References

[1] Mojtabai R Olfson M Sampson NA Jin R Druss B Wang PS Barriers to mental health treatment: results from the National Comorbidity Survey Replication. Psychol Med. (2011) 41(8):1751–61. 10.1017/S0033291710002291 21134315 PMC3128692

[2] Pepin R Segal DL Coolidge FL. Intrinsic and extrinsic barriers to mental health care among community-dwelling younger and older adults. Aging Ment Health. (2009) 13(5):769–77. 10.1080/13607860902918231 19882416

[3] Graham AK Weissman RS Mohr DC. Resolving key barriers to advancing mental health equity in rural communities using digital mental health interventions. JAMA Health Forum. (2021) 2(6):e211149. 10.1001/jamahealthforum.2021.1149 36218752 PMC11650711

[4] Aboujaoude E Gega L Parish MB Hilty DM. Editorial: digital interventions in mental health: current status and future directions. Front Psychiatry. (2020) 11:1–2. 10.3389/fpsyt.2020.00111 32174858 PMC7056878

[5] Cunningham JM Suldo SM. Accuracy of teachers in identifying elementary school students who report at-risk levels of anxiety and depression. School Ment Health. (2014) 6(4):237–50. 10.1007/s12310-014-9125-9

[6] Himle JA Weaver A Zhang A Xiang X. Digital mental health interventions for depression. Cogn Behav Pract. (2022) 29(1):50–9. 10.1016/j.cbpra.2020.12.009

[7] Graham AK Lattie EG Powell BJ Lyon AR Smith JD Schueller SM Implementation strategies for digital mental health interventions in health care settings. Am Psychol. (2020) 75(8):1080–92. 10.1037/amp0000686 33252946 PMC7709140

[8] Schueller SM Torous J. Scaling evidence-based treatments through digital mental health. Am Psychol. (2020) 75(8):1093–104. 10.1037/amp0000654 33252947 PMC7709142

[9] Balcombe L Leo DD. Digital mental health challenges and the horizon ahead for solutions. JMIR Ment Health. (2021) 8(3):e26811. 10.2196/26811 33779570 PMC8077937

[10] Borghouts J Eikey E Mark G De Leon C Schueller SM Schneider M Barriers to and facilitators of user engagement with digital mental health interventions: systematic review. J Med Internet Res. (2021) 23(3):e24387. 10.2196/24387 33759801 PMC8074985

[11] Torous J Roberts LW. Needed innovation in digital health and smartphone applications for mental health: transparency and trust. JAMA Psychiatry. (2017) 74(5):437–8. 10.1001/jamapsychiatry.2017.0262 28384700

[12] Nahum-Shani I Shaw SD Carpenter SM Murphy SA Yoon C. Engagement in digital interventions. Am Psychol. (2022) 10.1037/amp0000983 35298199 PMC9481750

[13] Ganapathy A Clough BA Casey LM. Organizational and policy barriers to the use of digital mental health by mental health professionals. Telemed E-Health. (2021) 27(12):1332–43. 10.1089/tmj.2020.0455 33646057

[14] Nicholas J Larsen ME Proudfoot J Christensen H. Mobile apps for bipolar disorder: a systematic review of features and content quality. J Med Internet Res. (2015) 17(8):e4581. 10.2196/jmir.4581 PMC4642376 26283290

[15] Merikangas KR Akiskal HS Angst J Greenberg PE Hirschfeld RM Petukhova M Lifetime and 12-month prevalence of bipolar spectrum disorder in the national comorbidity survey replication. Arch Gen Psychiatry. (2007) 64(5):543–52. 10.1001/archpsyc.64.5.543 17485606 PMC1931566

[16] Bauer M Kilbourne A Greenwald D Ludman E. Overcoming bipolar disorder: A comprehensive workbook for managing your symptoms and achieving your life goals. Oakland, CA: New Harbinger Publications (2009).

[17] Kilbourne AM Goodrich DE Lai Z Clogston J Waxmonsky J Bauer MS. Life goals collaborative care for patients with bipolar disorder and cardiovascular disease risk. Psychiatr Serv. (2012) 63(12):1234–8. 10.1176/appi.ps.201100528 23203358 PMC4132840

[18] Ryan KA Smith SN Yocum AK Carley I Liebrecht C Navis B The life goals self-management mobile app for bipolar disorder: consumer feasibility, usability, and acceptability study. JMIR Form Res. (2021) 5(12):e32450. 10.2196/32450 34898452 PMC8713087

[19] Kroenke K Spitzer RL Williams JB. The PHQ-9: validity of a brief depression severity measure. J Gen Intern Med. (2001) 16(9):606–13. 10.1046/j.1525-1497.2001.016009606.x 11556941 PMC1495268

[20] Mossman SA Luft MJ Schroeder HK The Generalized Anxiety Disorder 7-item scale in adolescents with generalized anxiety disorder: signal detection and validation. Ann Clin Psychiatry Off J Am Acad Clin Psychiatr. (2017) 29(4):227–234A. PMID: ; PMCID: 29069107 PMC5765270

[21] Bauer MS Vojta C Kinosian B Altshuler L Glick H. The Internal State Scale: replication of its discriminating abilities in a multisite, public sector sample. Bipolar Disord. (2000) 2(4):340–6. 10.1034/j.1399-5618.2000.020409.x 11252648

[22] McInnis MG Assari S Kamali M Ryan K Langenecker SA Saunders E Cohort profile: The Heinz C. Prechter longitudinal study of bipolar disorder. Int J Epidemiol. (2018) 47(1):28–28n. 10.1093/ije/dyx229 29211851 PMC5837550

[23] Karen H Sandra J. Contextual inquiry: a participatory technique for system design. In: Schuler D, Namioka A. (editors) Participatory design. Boca Raton, FL: CRC Press (1993). p. 177–210

[24] Hanington B Martin B. Universal methods of design - 125 ways to research complex problems, develop innovative ideas and design effective solutions. Beverly, MA: Rockport Publishers (2019).

[25] Flood M Ennis M Ludlow A Sweeney FF Holton A Morgan SC Research methods from human-centered design: potential applications in pharmacy and health services research. Res Soc Adm Pharm. (2021) 17(12):2036–43. 10.1016/j.sapharm.2021.06.015 34229952

[26] Le T Chi NC Chaudhuri S Thompson HJ Demiris G. Understanding older adult use of data visualizations as a resource for maintaining health and wellness. J Appl Gerontol. (2018) 37(7):922–39. 10.1177/0733464816658751 27401438

[27] Joe J Chaudhuri S Le T Thompson H Demiris G. The use of think-aloud and instant data analysis in evaluation research: exemplar and lessons learned. J Biomed Inform. (2015) 56:284–91. 10.1016/j.jbi.2015.06.001 26071683 PMC4532606

[28] Juniper EF. Validated questionnaires should not be modified. Eur Respir J. (2009) 34(5):1015–7. 10.1183/09031936.00110209 19880615

[29] Committee on the Learning Health Care System in America, Institute of Medicine. In: Smith M Saunders R Stuckhardt L McGinnis JM, editors. Best care at lower cost: the path to continuously learning health care in America. Washington DC: National Academies Press (US) (2013). Available from: http://www.ncbi.nlm.nih.gov/books/NBK207225/ (Accessed August 30, 2022). 24901184

[30] Gremyr A Malm U Lundin L Andersson AC. A learning health system for people with severe mental illness: a promise for continuous learning, patient coproduction and more effective care. Digit Psychiatry. (2019) 2(1):8–13. 10.1080/2575517X.2019.1622397

[31] Dixon L. Directions for future patient-centered and comparative effectiveness research for people with serious mental illness in a learning mental health care system. Schizophr Bull. (2014) 40(Suppl 1):S1–S94. 10.1093/schbul/sbt170 24489078 PMC3911266

[32] Nahum-Shani I Smith SN Spring BJ Collins LM Witkiewitz K Tewari A Just-in-time adaptive interventions (JITAIs) in Mobile health: key components and design principles for ongoing health behavior support. Ann Behav Med. (2018) 52(6):446–62. 10.1007/s12160-016-9830-8 27663578 PMC5364076

[33] Coppersmith D Dempsey W Kleiman EM Bentley KH Murphy SA Nock MK. Just-in-time adaptive interventions for suicide prevention: promise, challenges, and future directions. Psychiatry (2022) 1–17. 10.1080/00332747.2022.2092828 PMC9643598 35848800

[34] Wang L Miller LC. Just-in-the-moment adaptive interventions (JITAI): a meta-analytical review. Health Commun. (2020) 35(12):1531–44. 10.1080/10410236.2019.1652388 31488002

[35] Torous J Bucci S Bell IH Kessing LV Faurholt-Jepsen M Whelan P The growing field of digital psychiatry: current evidence and the future of apps, social media, chatbots, and virtual reality. World Psychiatry Off J World Psychiatr Assoc WPA. (2021) 20(3):318–35. 10.1002/wps.20883 PMC8429349 34505369

